# Anaphylaxis to Pfizer/BioNTech mRNA COVID-19 Vaccine in a Patient With Clinically Confirmed PEG Allergy

**DOI:** 10.3389/falgy.2021.715844

**Published:** 2021-09-29

**Authors:** Morgan D. McSweeney, Manoj Mohan, Scott P. Commins, Samuel K. Lai

**Affiliations:** ^1^Mucommune, LLC, Durham, NC, United States; ^2^Okemos Allergy Center, Okemos, MI, United States; ^3^Division of Allergy, Immunology and Rheumatology, Department of Medicine, University of North Carolina—Chapel Hill, Chapel Hill, NC, United States; ^4^Division of Pharmacoengineering and Molecular Pharmaceutics, Eshelman School of Pharmacy, University of North Carolina—Chapel Hill, Chapel Hill, NC, United States; ^5^Department of Microbiology and Immunology, University of North Carolina—Chapel Hill, Chapel Hill, NC, United States; ^6^Department of Biomedical Engineering, University of North Carolina—Chapel Hill, Chapel Hill, NC, United States

**Keywords:** vaccine allergy, vaccine adverse event, anaphylaxis, polyethylene glycol, anti-PEG antibodies, liposome, mRNA vaccine, COVID-19 vaccine

## Abstract

Although allergic responses to the mRNA COVID-19 vaccines are rare, recent reports have suggested that a small number of individuals with allergy to polyethylene glycol (PEG), a component of the mRNA lipid nanoshell, may be at increased risk of anaphylaxis following vaccination. In this report, we describe a case of a patient who received an mRNA COVID-19 vaccine, experienced anaphylaxis, and was subsequently confirmed to have anti-PEG allergy by skin prick testing. The patient had previously noticed urticaria after handling PEG powder for their occupation and had a history of severe allergic response to multiple other allergens. Importantly, as many as 70% of people possess detectable levels of anti-PEG antibodies, indicating that the detection of such antibodies does not imply high risk for an anaphylactic response to vaccination. However, in people with pre-existing anti-PEG antibodies, the administration of PEGylated liposomes may induce higher levels of antibodies, which may cause accelerated clearance of other PEGylated therapeutics a patient may be receiving. It is important to improve awareness of PEG allergy among patients and clinicians.

Polyethylene glycol (PEG) is a hydrophilic polymer incorporated in the form of lipid-PEG conjugates in both of the mRNA COVID-19 vaccines from Pfizer/BioNTech and Moderna to stabilize the lipid nanoparticles carrying the mRNA (1–3). PEG is routinely used in the formulation of protein drugs and nanomedicines to reduce aggregation and improve distribution and elimination kinetics (4–8). Allergic reactions to PEG have been noted for several PEGylated drugs, leading to adverse events ranging from infusion reactions to anaphylaxis (9–13). There have also been numerous reports of allergic responses to PEGs present in a diverse group of goods, ranging from osmotic laxatives to soaps, lotions, and cosmetics, as well as to other pharmaceuticals such as analgesics, depo injections, laxatives, and various tablet formulations of drugs such as antacids and antibiotics, which may include PEG as an excipient (14–17).

Since allergic reactions to vaccines are exceptionally rare [~1.3 cases per million doses (18)], when several cases of anaphylaxis to the Pfizer/BioNTech and Moderna vaccines were noted soon after rollout, there were speculations that the response might be attributed to PEG sensitivity (19–21). The link between anaphylaxis to the vaccines and PEG-hypersensitivity was recently reported for one individual in the U.K. (22). Here, we report another case of an individual with a PEG-allergy that was identified after experiencing anaphylaxis to the Pfizer/BioNTech COVID-19 vaccine.

In February 2021, the patient, a 20-year-old female, presented to an emergency room via emergency medical services due to anaphylaxis following administration of the Pfizer/BioNTech COVID-19 vaccine. Approximately 10 min after vaccination, she developed headache and elevated heart rate, which she attributed to feelings of anxiety regarding vaccination. However, 5 min later, the patient noted having angioedema and cramping abdominal pain. She self-administered intramuscular epinephrine and oral diphenhydramine, and emergency medical services were notified.

The patient's past medical history included allergic and hypersensitivity reactions to foods, moderate persistent asthma, atopic dermatitis, seasonal and perennial allergic rhinitis, attention deficit hyperactivity disorder, and obesity (BMI 45.2 kg/m^2^). Specifically, the patient had previously experienced anaphylaxis to yeast, buckwheat, cantaloupe, as well as serious, but non-anaphylactic, reactions to corn. The patient was also allergic to feathers, pet dander, dust mite, grass, and trees. The patient reported sensitivity to scented cosmetics and soaps, some of which led to urticaria.

The patient had previously noticed topical allergic reactions to an oral osmotic laxative, polyethylene glycol 3350 Da (PEG 3350). Upon preparation of the oral osmotic laxative for her occupation (as a residential technician working with adults with developmental disabilities), she had skin contact with small amounts of PEG 3350 powder and developed significant local urticaria, which occurred on at least 3 separate occasions. However, despite these prior events, at the time of COVID vaccine administration, the patient was not aware of a potential allergy to PEG.

## Medications

The patient regularly takes dextroamphetamine and amphetamine, fluoxetine, olopatadine hydrochloride nasal spray, beclomethasone diproprionate HFA inhaler, fluticasone nasal spray, montelukast, levocetirizine dihydrochloride, and loratadine as needed. Among these, the fluticasone nasal spray contains polysorbate-80, and levocetirizine dihydrochloride contains PEG 400 Da. The patient has not previously noticed hypersensitivity to either medication. It should be noted that prior work has shown that at least some APA molecules require a PEG epitope that spans ~12–16 repeat ethylene oxide units (23), which translates to PEG MW generally in excess of ~500–700 Da. However, SPT testing with PEGs as small as 300 Da have shown occasional positive responses (14).

## Emergency Department and Hospital Course and Follow-Up

In the emergency department, patient had worsening gastrointestinal symptoms, including emesis. An additional dose of epinephrine was administered. No serum tryptase was taken in the acute phase. The patient was admitted for observation overnight. On the day of discharge, the patient's gastrointestinal symptoms had resolved, although she continued to experience headache, dizziness, and generalized weakness. The patient was prescribed a 5-day course of prednisone.

The patient was seen by their allergy and immunology specialist ~3.5 weeks after vaccination, who performed a skin prick test (SPT) to assess hypersensitivity to PEG 3350 and polysorbate-80. Polysorbate 80 contains PEG moieties and has been reported to be cross reactive in some patients with PEG hypersensitivity (16). The patient did not take antihistamines for 1 week prior to SPT. A 1:10 solution of PEG 3350 was prepared for SPT by diluting 17 g PEG 3350 in 8 ounces of water (i.e., the preparation of a dose of Miralax laxative) and then adding 1 part of that solution to 9 parts saline. Upon PEG SPT, the patient had a ~8–10 mm wheal and ~8–10 mm flare. A polysorbate-80 solution was prepared for SPT using a stock of polysorbate-80 solution (compound #62 on the NAC80 tray). Upon polysorbate-80 SPT, the patient had a ~5–10 mm wheal and flare.

## Discussion

This report describes anaphylaxis to the Pfizer/BioNTech COVID-19 vaccine in a patient who was later confirmed to be allergic to polyethylene glycol. This report adds to several other recent publications describing PEG allergy-related reactions to mRNA COVID-19 vaccines (22, 24, 25), supporting the need for individuals with suspected allergy to PEG to consult an allergist prior to vaccination. Individuals with a history of anaphylactoid reactions to several different types of drugs should raise suspicion for allergy to excipients that may be common across formulations, such as PEG.

One recent report describing a patient who experienced anaphylaxis following receipt of the Pfizer-BioNTech vaccine confirmed PEG sensitivity via basophil activation test (with positive signal upon stimulation of whole blood *in vitro* with PEG 4000 at 0.2 mg/mL) (24). However, a prior study including 10 PEG-sensitive patients and 16 controls demonstrated that *in vitro* tests have poor sensitivity for detection of PEG allergy if not conducted within a few months of a reaction and cannot be used to rule-out PEG allergy (14). To minimize the risk of causing a serious systemic reaction through SPT with high concentration, high MW PEG, and to also reduce risk of false negative findings, it has been suggested that PEG SPT should be conducted in a stepwise fashion, starting with lower MW PEGs (300, 2000, 3000 Da), increasing up to 20,000 Da, and then stepwise increasing the concentration of PEG used for SPT (14). Given the noted risks of systemic reactions from PEG SPT, such testing should be performed in a setting well-equipped to manage immediate-type allergic reactions. That same report found that some patients lose reactivity to PEG SPT over time, starting with loss of SPT result to lower MW PEGs (~3,000–6,000 Da), but maintain reactivity on SPT to higher MW PEGs (~20,000 Da). This presents the risk that a diagnosis of PEG allergy may be missed if a single concentration of low MW PEG is used for testing. Indeed, a recent report of a patient who experienced anaphylaxis following SARS-CoV-2 vaccination showed negative SPT results with PEG 2000 at 0.1% and with residue from the vaccine vial. However, SPT was repeated with a higher concentration of PEG 4000 (1%) and triggered anaphylaxis (22). In that patient, SPT only with PEG 2000 at 0.1% prior to vaccination would have failed to indicate risk of hypersensitivity.

We are routinely exposed to PEG in daily settings due to the broad inclusion of PEG in hygiene products (e.g., toothpastes, shampoo, soap), skincare products (e.g., body lotion, cosmetics) and processed foods, among other things (16, 26). Very large doses of PEG are used as laxatives in children and adults and as part of bowel preparations prior to colonoscopy (27). Despite this, diagnoses of PEG allergy remain rare, which is consistent with the still very low rates of anaphylaxis and injection site reactions with both Pfizer/BioNTech and Moderna vaccines. Nevertheless, given that the large Phase 3 vaccine trials for the mRNA vaccines were restricted to patients without a prior history of allergy to any vaccine components (including PEG), the actual rates of anaphylactic responses to the COVID-19 vaccines in this subpopulation remains not well-understood. Not surprisingly, since rollout, patients with a prior history of allergy to vaccine components have been advised to choose a different vaccine option.

We emphasize here that both Pfizer/BioNTech and Moderna vaccines are exceptionally safe in the vast majority of individuals, with the benefits far outweighing any possible downsides, despite rare allergic responses such as the one in this report. In light of the severe long-term health consequences of COVID-19 observed even in some recovered patients (28–32), the small risk of PEG allergy (which can be readily cared for when it occurs) is justifiable. It should be further noted that the majority of the population already possesses low levels of APA of varying isotypes [perhaps >70% of people (33)]. Thus, the mere presence of pre-existing APA (particularly low titers of APA) does not imply appreciably greater risks for anaphylactic response to vaccination. Instead, it is likely that only the small number of individuals with very high titers of APA are at elevated risks for PEG allergy. However, in people with pre-existing APA, administration of PEGylated liposomes may induce the production of higher levels of APA, as seen with various animal studies (26). It remains to be determined whether the vaccines may induce elevated levels of anti-PEG antibodies (APA: IgE, IgG, or IgM) that, in turn, could theoretically increase the risks of adverse reactions or loss of efficacy in a subset of individuals who are also treated with other PEGylated medicines. Given the large fraction of the population with low levels of preexisting APA that may predispose them for induction of higher titers of APA, we believe this is a question that should be rigorously investigated going forward.

It might be expected that PEG sensitivity to the first dose of mRNA vaccine might predispose similar reactions to additional doses of the vaccine, raising concern for booster shots intended to enhance immunity against viral variants. However, a recent case series of patients who experienced anaphylaxis at their first mRNA vaccination found that 19 of 19 patients who still chose to receive the second dose did not experience anaphylaxis on the second dose, possibly suggesting a role for an allergic, but non-IgE-mediated mechanism, such as mast-cell activation or complement activation to a lipid or PEG component of the vaccine, as has been occasionally observed with other PEGylated lipid nanoparticles (34, 35). Determining the titers of isotypes of APA in these individuals would allow us to better understand whether circulating APA may be an effective marker for predicting PEG sensitivity.

Currently, screening for PEG allergy at the time of vaccination remains prohibitively challenging to perform and of uncertain benefit/cost ratio, given the low rates of anaphylactic reactions observed to date and generally good outcomes among patients who experience anaphylaxis. It remains to be determined whether APA levels could predict potential allergic responses to mRNA vaccines formulated with PEGylated lipids. Finally, it may be important to improve awareness of PEG allergy among pharmacists and physicians, as well as awareness of which medications contain PEG (36), as PEGylated therapies are used with increasing frequency in the clinical setting. This is more relevant now than ever; more individuals have been treated with a PEGylated intervention (namely, the Pfizer/BioNTech and Moderna COVID-19 vaccines) in the past year than in the 25+ year history of use of all prior PEGylated therapies combined.

## Data Availability Statement

The original contributions presented in the study are included in the article/supplementary material, further inquiries can be directed to the corresponding author/s.

## Ethics Statement

Ethical review and approval was not required for the study on human participants in accordance with the local legislation and institutional requirements. Written informed consent for participation was not required for this study in accordance with the national legislation and the institutional requirements.

## Author Contributions

All authors listed have made a substantial, direct and intellectual contribution to the work, and approved it for publication.

## Funding

This work was supported by The David and Lucile Packard Foundation (2013-39274, SL), National Institutes of Health (R01 HL141934; SL), and Eshelman Institute of Innovation (SL).

## Conflict of Interest

MDM and SL are inventors on Intellectual Property (IP) related to methods to overcome anti-PEG antibodies. This IP has not been licensed. MDM is employed by Mucommune LLC. SL and SC are employed by UNC-Chapel Hill. SC has received compensation from Genentech (speaker's bureau) and UpToDate (author royalties). The remaining author declares that the research was conducted in the absence of any commercial or financial relationships that could be construed as a potential conflict of interest.

## Publisher's Note

All claims expressed in this article are solely those of the authors and do not necessarily represent those of their affiliated organizations, or those of the publisher, the editors and the reviewers. Any product that may be evaluated in this article, or claim that may be made by its manufacturer, is not guaranteed or endorsed by the publisher.
